# An Interesting Case: Sunitinib-Induced Microangiopathic Hemolytic Anemia and Nephrotic Syndrome

**DOI:** 10.4274/tjh.galenos.2020.2020.0532

**Published:** 2021-06-01

**Authors:** Veysel Haksöyler, Semra Paydaş

**Affiliations:** 1 Private Adana Medline Hospital, Adana, Turkey; 2 Çukurova University Faculty of Medicine, Department of Oncology, Adana, Turkey

**Keywords:** Sunitinib, Nephrotic syndrome, Hemolytic anemia, Microangiopathic hemolytic anemia

## To the Editor,

Sunitinib is a heterodimeric oral tyrosine kinase inhibitor that targets a large number of receptors, including VEGFR and PDGFR. Anti-VEGF treatments can cause hypertension, proteinuria, neutropenia, anemia, and thrombocytopenia [1]. It has been shown in animal experiments that vascular endothelial growth factor (VEGF) contributes to the repair of glomerular endothelium in experimental microangiopathia and anti-VEGF antibodies cause proteinuria by glomerular dissociation and downregulation of nephrin receptors [2]. The increase of VEGF levels in the blood 2-3 weeks after thrombotic microangiopathy (TMA) supports the idea of VEGF-mediated repair of the glomerular endothelium [3]. Anti-VEGF treatment may cause thrombosis due to the procoagulant phospholipids released as a result of the disruption of plasma membrane integrity and due to the decrease in the levels of nitric oxide and prostaglandin I2, which contributes to the production of VEGF [4].

**Figure 1:**
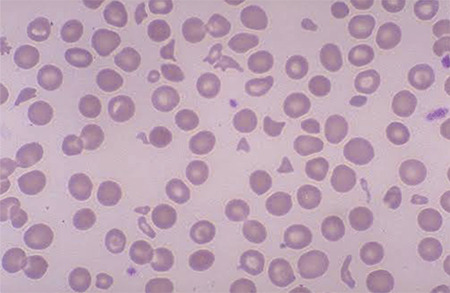
Peripheral blood smear showed rare schistocytes and mild thrombocytopenia.

A 54-year-old woman was receiving sunitinib for a metastatic gastrointestinal stromal tumor (GIST). She presented to the clinic 8 months after the initiation of therapy with microangiopathic hemolytic anemia (MAHA) and nephrotic syndrome (NS). Proteinuria (3.5 g) was detected in the 24-h urine collection. The platelet count was 35000/mm^3^, white blood cell count was 6700/mm^3^, and hemoglobin was 7 g/dL. In the blood smear, normochromic normocytic anemia, diffuse schistocytes, and fragmented erythrocytes were present (Figure 1). Sunitinib was discontinued and methylprednisone was started with the resolution of symptoms. MAHA and NS relapsed with re-challenge with sunitinib. Symptoms resolved after the discontinuation of sunitinib.

Bollee et al. published the case of a patient with malignant skin hidradenoma who developed hypertension and proteinuria. Renal biopsy showed microangiopathic anemia [5]. A second reported case involved metastatic renal cell carcinoma; hypertension, nephrotic proteinuria, azotemia, creatinine increase, oliguria, thrombocytopenia, and anemia developed and kidney biopsy showed focal segmental glomerulosclerosis and TMA. After the cessation of sunitinib, the patient recovered [6]. A third case involved hypertension and proteinuria; a kidney biopsy showed TMA. This patient improved with the cessation of sunitinib and steroids [7]. In a fourth case of metastatic GIST, the patient presented with hypertension, loss of vision, seizures, anemia, thrombocytopenia, acute renal failure, and posterior leukoencephalopathy with schistocytes in the blood smear. After the cessation of sunitinib, he improved [8]. A fifth case involved metastatic renal cell carcinoma with nephrectomy as well as nephrotic proteinuria. TMA was confirmed by kidney biopsy. Kidney functions and proteinuria almost entirely improved after stopping sunitinib and starting steroids [9]. In another case of metastatic renal cell carcinoma, three weeks after the start of sunitinib, hypertension, proteinuria, thrombocytopenia, and anemia developed. Schistocytes were noticed in the blood smear. The patient’s symptoms improved after the discontinuation of sunitinib [10].

In contrast to many cases discussed, the case that we present here was not a case of renal cell carcinoma but rather metastatic GIST. Generally, sunitinib is used in the first line of treatment for renal cell carcinoma, but it is used after imatinib in GIST treatment, as we did for this patient. Therefore, it is interesting that this toxicity developed after the second tyrosine kinase inhibitor. In this regard, it is an infrequent phenomenon. Similar to other patients mentioned, hypertension was detected in our patient before the development of toxicity. As in most of the other cases, our patient’s condition improved almost completely after stopping sunitinib. Our patient did not undergo a kidney biopsy because she had thrombocytopenia and therefore rejected the kidney biopsy. Furthermore, the diagnosis was made clinically, so a biopsy was not required.

The use of anti-VEGF drugs has become widespread and there are limited published data about such severe toxicities (Table 1). For this reason, we wanted to present this rare case that we found and we believe that it can contribute to the literature.

## Figures and Tables

**Table 1:**
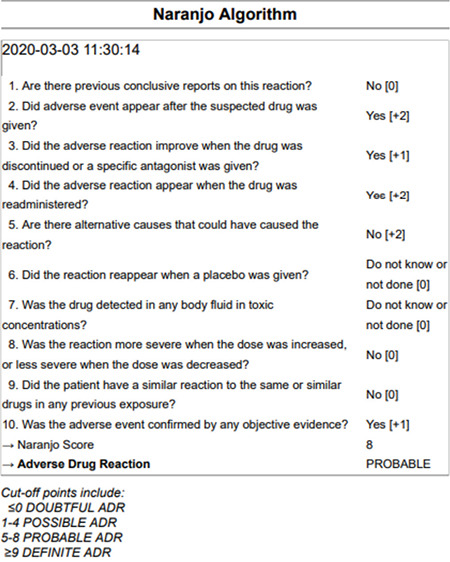
Naranjo algorithm assessment.
